# Structural Chromosome Abnormalities Associated with Obesity: Report of Four New subjects and Review of Literature

**DOI:** 10.2174/138920211795677930

**Published:** 2011-05

**Authors:** Majed J Dasouki, Erin L Youngs, Karine Hovanes

**Affiliations:** 1 Departments of Pediatrics and Internal Medicine, Kansas University Medical Center, Kansas City, Kansas, USA; 2 Departments of Pediatrics and Internal Medicine, Kansas University Medical Center, Kansas City, Kansas, USA; 3 Departments of Psychiatry & Behavioral Sciences and Pediatrics, Kansas University Medical Center, Kansas City, Kansas, USA; 4 CombiMatrix Diagnostics, Irvine, California, USA

**Keywords:** Obesity, aCGH, chromosome, deletion, duplication, translocation, fluorescent in situ hybridization, CNV.

## Abstract

Obesity in humans is a complex polygenic trait with high inter-individual heritability estimated at 40–70%. Candidate gene, DNA linkage and genome-wide association studies (GWAS) have allowed for the identification of a large set of genes and genomic regions associated with obesity. Structural chromosome abnormalities usually result in congenital anomalies, growth retardation and developmental delay. Occasionally, they are associated with hyperphagia and obesity rather than growth delay. We report four new individuals with structural chromosome abnormalities involving 10q22.3-23.2, 16p11.2 and Xq27.1-q28 chromosomal regions with early childhood obesity and developmental delay. We also searched and summarized the literature for structural chromosome abnormalities reported in association with childhood obesity.

## INTRODUCTION

Obesity is a major growing health problem facing children and adults worldwide and particularly in the Westernized world. It represents an energy imbalance in which energy intake exceeds expenditure. While decreased physical activity and increased caloric intake are the major forces behind this epidemic, there is strong evidence supporting the role of major genes and minor genomic variants in causing or contributing to human obesity. Various genetic techniques had been used to identify the genetic basis of severe early childhood obesity and with the more common forms of obesity usually seen in older individuals. To date, cytogenetic studies followed by appropriate molecular analysis in a small group of children with severe childhood obesity have shown specific structural chromosome abnormalities. Large cohorts of obese adults have also been studied using genome-wide linkage and association scans. In most of these studies, single nucleotide polymorphism (SNP) based platforms were used rather than copy number variants. Beginning in 1996, the “Human Obesity Gene Map” provided a catalogue of all known genetic variants and chromosomal loci associated with or linked to obesity-related traits [1]. The most recent update was published in 2006 [2] and included 176 single-gene mutations in 11 different genes, 50 loci related to known Mendelian syndromes, 244 murine adiposity related genes, 408 animal model based quantitative trait loci (QTLs) and 253 human QTLs from 61 genome-wide scans.

In this report, we present our experience in a small series of individuals with childhood obesity and other health problems presenting for genetic services. Four individuals with obesity and other health problems were found to have structural chromosome abnormalities. We briefly review structural chromosome abnormalities associated with obesity reported in the literature. 

## SUBJECTS AND METHODS

### Clinical Reports

#### Subject 1

**Fig. (1):**
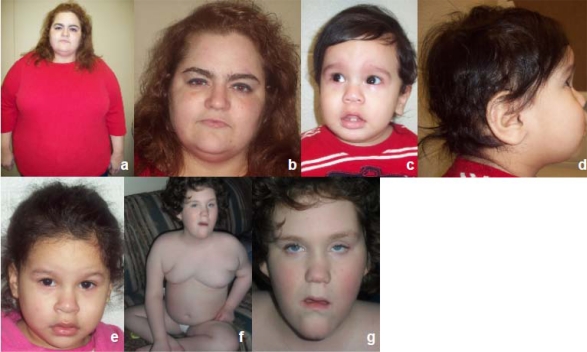
Photographs of Subjects 1-4. Subject 1 (**a,b**) has synophrys, obesity and urticaria (not shown). Subject 2 (**c,d**) and his sister, Subject 3 (**e**) have a prominent forehead, bulky ears, micrognathia and full lips. Subject 4 (**f,g**) has myopathic facies, ptosis, long and narrow face, truncal obesity, a prominent forehead and prognathism.

At 34 years of age, this woman (Fig. **1a**,**1b**) presented with obesity since early childhood and developed idiopathic urticaria which became prednisone dependent. Therefore, multiple hives and target lesions were noted. 

She was diagnosed with endometriosis at 22 years of age and a large lipoma on her upper back required resection at 34 years of age. She had recurrent facial methicillin resistant *Staphylococcus aureus* (MRSA) infection and poison ivy. She had no cognitive abnormalities. An abdominal CT scan performed at 35 years of age showed a normal gastrointestinal tract but unexpectedly marked atrophy of the right kidney was seen with bilateral renal focal clubbing and cortical scarring. Calcified renal cysts were also noted in the right kidney. Extensive laboratory workup (rheumatoid factor, aldosterone, cortisol, FSH/LH, insulin, testosterone, celiac disease antibody panel, Hepatitis B antibody profile, ferritin, *H. pylori* profile, serum protein electrophoresis, thyroid peroxidase antibody, anti-thyroglobulins and histamine and tryptase levels) were all within normal range. Her serum uric acid was mildly increased and an ANA screen was positive at a low titer (1 in 40, speckled pattern). Her weight was 133.36 kg (>95^th^ centile), height was 155 cm and BMI was 55.5 kg/m^2^. She had synophrys, a “buffalo hump” fat pattern and a recurring large lipoma on her upper back region. 

**Fig. (2):**
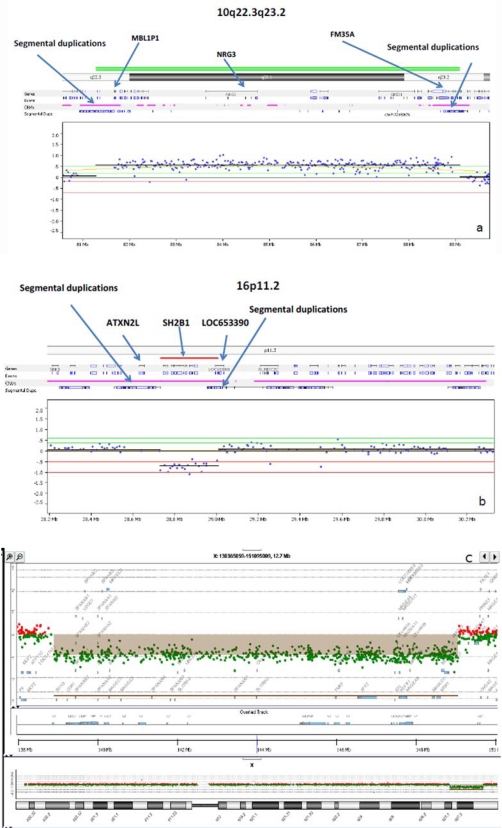
Array CGH (log2 R) profiles for Subjects 1-4. Subject 1(**a**) has a large 7.8 Mb duplication found at 10q22.3-q23.2 between the segmental LCR3 and LCR4 regions (arrows). Subjects 2 and 3 (**b**) have a small maternally inherited 16p11.2 deletion found which lies within a region of segmental duplications with location of representative genes (*ATXN2L, SH2B1 and LAT*) noted. Subject 4 (**c**) has a large 10.69 Mb deletion at Xq27.1-q28. The deleted X chromosome region detected by the array is shown above the X chromosome ideogram.

**Fig. (3):**
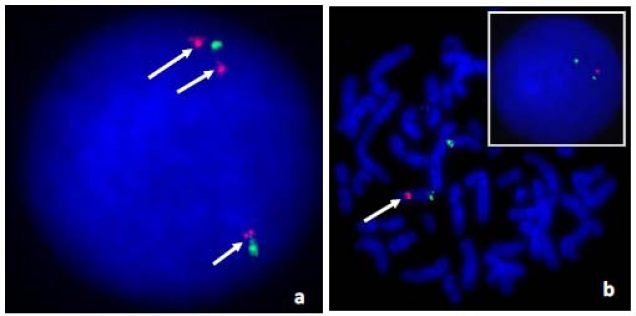
Confirmatory fluorescent in situ hybridization (FISH) studies in Subjects 1, 2, and 3. In Subject 1 (**panel a**), the 10q23.2 (RP11-830J13) Rhodamine labeled BAC clone showed 3 copies (arrows) while the “control” chr. 10p11.21 FITC labeled BAC clone (RP11-876K20) showed normal hybridization pattern in interphase nuclei. Subjects 2, 3 and their mother shared the same 16p11.2 deletion (**panel b**) detected by the RP11-1136I3 (Rhodamine) BAC clone (arrow). The RP11-133L7 (FITC) BAC clone was the control probe.

A 105K-whole-genome array comparative genomic hybridization (aCGH) study revealed a 7.8 Mb duplication at chromosome 10q22.3-q23.2 (Fig. **2a**) (base position: 81,281,895-89, 091,213; hg18) which was absent in her mother. Her father was not available for evaluation. The duplication was confirmed by fluorescent in situ hybridization (FISH) using the BAC clone “RP11-830JJ13” (Fig. **3a**). 

#### Subjects 2 and 3

At 8 months of age, this Hispanic boy (Fig. **1c**,**1d**) was evaluated for a suspected diagnosis of Prader-Willi syndrome because of his rapid weight gain and developmental delay. He was born at term and his mother had gestational diabetes mellitus. He had feeding difficulties for the first four months of life followed by developmental delays. His weight was 11.53 kg (>95^th^ centile), length was 73.5 cm (50-75^th^ centile) and BMI was 21.3 kg/m^2^ with a head circumference of 46.25 cm (75-90^th^ centile). Frontal bossing was noted with moderate micrognathia, almond-shaped eyes, flat-nasal bridge, small appearing hands with distal tapering of fingers, deep palmar creases, one large café au lait spot on the left shoulder, undescended testes, and a head lag. DNA methylation testing for the Prader-Willi syndrome/Angelman syndrome region at chromosome 15q11.2 was normal (not shown). Genomic DNA oligo-array CGH analysis was then performed which showed a small 16p11.2 deletion (base position: 28,730,299-29,009,896; hg18) (Fig. **2b**) and confirmed by FISH analysis using the BAC clone “RP11-1136I3” (Fig. **3b**). 

His mother was 30 years old, weighed 58.2 kg and was 150.3 cm tall. Her BMI was 25.8 kg/m^2^. She reported no significant health problems. Her other child (subject 3), a 34 month old daughter with obesity and speech delay, presented for genetic evaluation. The pregnancy was complicated by preterm labor at 36 weeks gestation with a history of maternal syphilis and gestational diabetes mellitus. Birth weight was 2.43 kg (39^th^ centile), length 45.9 cm (46^th^ centile), BMI was 11.5 kg/m^2 ^and head circumference was 29.5 cm (6^th^ centile). Congenital syphilis was excluded. She walked at 12 months of age. At 34 months of age, her weight was 19.9 kg (>97^th^ centile), length was 97.5 cm (>95^th^ centile), BMI was 20.9 kg/m^2^ and head circumference was 50 cm (90^th^ centile). She presented with frontal bossing, one small café au lait spot on the lower back and delayed speech (Fig. **1e**). Her serum liver and lipid profiles, renal ultrasound and bone age were normal for her age. These family members also showed the same 16p11.2 deletion as subject 2.

#### Subject 4

At 8 years of age, this Caucasian girl was evaluated and found to have a *de novo* germline deletion involving chromosome Xq27.1-q28 region by standard chromosome analysis (not shown). This chromosome abnormality was confirmed by aCGH analysis and a 10.69 Mb deletion was found (base position: 139,354,859-150,046,723; hg18) (Fig. **2c**). She was the product of a 37 weeks gestation complicated by maternal tobacco smoking. Delivery was spontaneous vaginal and birth weight was 2.41 kg. Postnatal health problems included a slow weight gain, hypotonia, microcephaly, global developmental delays, right eye esotropia, and chronic constipation. A brain CT scan, thyroid function, quantitative plasma amino acid and urine organic acid profiles were all normal. She also had normal *MECP2* gene sequencing, *SNRPN* gene methylation testing for PWS and *FMR1* gene methylation with the CGG repeat determination at 20 indicating one allele. Quantitative urine mucopolysaccharides levels were elevated at 20.8 (<12.0 mg/mmoL Cr). Her weight was 33.7 kg (90-95^th^ centile), height was 100 cm (<5^th^ centile), head circumference was 50 cm, and BMI 33.7 was kg/m2. She was dysmorphic (myopathic facies, dolicho-microcephaly, almond-shaped eyes, narrow, long face and nose, pinched nostrils, prominent philtrum, small mouth, small ear lobes, and a narrow palate). She had truncal obesity, small hands and feet and severe speech and cognitive delays (Fig. **1f**,**1g**). She was treated with growth hormone.

### Molecular Cytogenetic Studies

In this study, four individuals were studied and found to have various chromosome aberrations associated with early onset obesity and other findings. Standard chromosome analysis of peripheral blood lymphocytes was initially performed in subject 4. DNA was isolated according to standard procedures and used for chromosome microarray studies. FISH analysis used genomic region specific BAC clones (Table **1**) for Subjects 1, 2 and 3 and obtained from The Center for Applied Genomics (TCAG, Toronto, Canada). Quantitative PCR amplification of an Xq27.1 gene was used in Subject 4 to confirm the cytogenetic deletion (not shown). Three separate Agilent Technologies (Santa Clara, CA) oligonucleotide CGH array platforms (105K platform in Subject 1, 180K platform in Subjects 2 and 3 and 244K platform in Subject 4) were used with genomic DNA isolated following manufacturer’s recommendations and as previously described [3,4]. Each sample was subjected to one hybridization experiment using Cy3 fluorescently labeled test DNA paired with Cy5 labeled reference DNA. Probe hybridization signals were expressed as the log_2_ ratio of signal intensities of a test sample versus signal intensities of a reference sample and array data were analyzed using the Nexus Copy Number™ software (Biodiscovery, El Segundo, CA) based on the genome content sourced from the UCSC hg18 human genome (NCBI build 36, March 2006). Array CGH studies for Subjects 1, 2 and 3 were performed at CombiMatrix^TM^ (Irvine, CA) while Subject 4 was studied at The Children’s Mercy Hospital Microarray Laboratory (Kansas City, MO). 

### Literature Search Discovery

To further identify structural chromosome abnormalities playing a role in the causations of obesity, we preformed an online PubMed search for structural chromosome abnormalities reported in the literature using key words such obesity, chromosome deletion, duplication, translocation, inversion, and the names of leading genetics journals. The results of this search were compiled in Table **2**. 

## RESULTS

The major clinical findings in our four individuals were summarized in Table **1** and the results of their molecular studies. While obesity is a shared clinical finding, each individual had a different complex phenotype due to their unrelated molecular genetic abnormality except for Subjects 2 and 3 who were siblings. The aCGH profiles for Subjects 1 through 4 are shown in Fig. (**2a-2c**) representing the regions of genomic abnormality. The regions recognized segmental duplications (Fig. **2a**,**2b**) known to mediate the copy number abnormalities of the chromosomes involved are highlighted. In Fig. (**3a**,**3b**), the duplication of chromosome 10q22.3-q23.2 and deletion of 16p11.2 were confirmed by FISH analysis. The BAC clones used for the FISH studies in each subject are shown in Table **1**. The parents of subject 4 had normal peripheral blood chromosomes studies. 

## DISCUSSION

In this group of four subjects, generalized or truncal obesity was a major clinical feature. Early feeding difficulties (Subjects 2 and 4), hypotonia (Subject 4) and developmental delays (Subjects 2 and 4) followed by rapid weight gain led to the clinical suspicion of Prader-Willi syndrome (PWS). The PWS diagnosis was largely excluded by the normal *SNRPN* gene methylation studies which ruled out genetic defects of the PWS/AS (Angelman syndrome) region. Subject 1 also presented as an adult with an unexplained complex phenotype which warranted a detailed genetic evaluation and revealed a chromosome 10q22.3-q23.2 duplication. Facial dysmorphism was noticeable in each of the four individuals but the most striking observations were seen in Subject 4. Developmental and speech delays were present in subjects 2, 3 and 4. 

Human DNA fragments >1 kb in size and of >90% DNA sequence identity have been termed low copy repeats (LCRs) or segmental duplications which can stimulate or mediate constitutional (i.e., inherited; both recurrent and non-recurrent) and somatic genomic rearrangements [5]. The advent and application of aCGH resulted in the identification of an increasing number of genomic disorders (microdeletion/microduplication syndromes) with nonallelic homologous recombination (NAHR) as the predominant underlying molecular mechanism using the flanking LCRs as recombination substrates [5]. Examples of common copy repeat loci involved in genomic disorders due to copy number abnormalities include 7q11.2, 15q11.2, 22q11.2 and others. The phenotypic consequences observed in these disorders may result from mechanisms such as deletions or duplications of dosage sensitive genes, position effect, unmasking of recessive mutations or functional polymorphisms of the remaining allele when a deletion occurs, or through effects of transvection (interaction between alleles on homologous chromosomes) [6,7]. 

The 10q22.3-q23.2 region is characterized by a complex set of low-copy repeats (LCRs 3, 4), which can give rise to various genomic changes mediated by nonallelic homologous recombination. Large duplications involving the 10q22-q23 region are very rare with only four cases reported overlapping this region [8-12]. However, six microduplications within the 10q22.3-q23.3 region have been reported recently by van Bon *et al. *[13]. Subject 1 in this report has a large 7.8 Mb duplication at 10q22.3-q23.2 which is between LCR 3 and 4. It is interesting to note that the *hsa-miRNA346* gene (human microRNA) lies within this duplicated region (base position: 88,022,451-88,026,545). Microdeletion/duplications involving mirRNA genes may add another level of complexity because of their multiple target genes. For example, miRNA346 has 72 predicted targets [miRBase, 2011]. Unlike the six individuals reported by van Bon *et al*. [13], Subject 1 has severe obesity and recurrent large lipomas requiring surgical resection. She is not known to have intestinal polyposis. The 10q22.3-q23.2 duplication region seen in this subject does not include the *PTEN* and *BMPR1* genes which cause Bannayan-Riley-Ruvalcaba syndrome (BRRS) and Juvenile Polyposis Syndrome (JPS), respectively. Lipomatosis is a known feature of BRRS. In addition, positive linkage with D10S535 (10q22.3; base position:76,240,707-76,440,904, hg19), and D10S1267 (10q24.32; chr10:104,278,612-104,478,824bp) and obesity phenotypes have been reported separately in both White and African-American individuals [14,15]. We speculate that our subject’s 10q duplication may have disrupted the expression of a dosage sensitive gene such as *PTEN* or another gene or non-coding signal in this region contributing to her recurrent lipomatosis and obesity. 

The clinical and molecular findings in Subjects 2 and 3 are consistent with the recently described 16p11.2 obesity- related microdeletion syndrome (position 28.7-28.9 Mb) [16-18]. We mapped this deletion to a region of segmental duplications at 16p11.2 and found it to be maternally inherited and confirmed by FISH analysis. This deletion encompasses the *SH2B1* gene which is implicated in murine obesity [19]. The mother of these two affected children appeared to be non-penetrant for this pathogenic copy number variant, a phenomenon known to occur. This locus appears to be distinct from the more proximal 16p11.2 (29.5-30.1 Mb) autism associated locus. 

Several subjects with contiguous gene deletions involving the *FMR1* gene locus have been reported and were comprehensively reviewed recently by Coffee *et al*. [20]. They cited a total of 71 deletions that were classified as small or large deletions. Subject 4 who has severe developmental delays, facial dysmorphism and truncal obesity was found to carry a *de novo* 10.69 Mb deletion at Xq27.1-q28 (Fig. **2c**). Major genes within the deleted region include *FMR1, FMR2, IDS, MTM1* and *MTMR1*. In addition to Hunter syndrome and myotubular myopathy, other phenotypic abnormalities noted in other individuals with contiguous gene deletions including the *FMR1* are obesity [21,22,24], cherubism [23], overgrowth [25,26], and macrocephaly [27,28]. A Prader-Willi syndrome-like phenotype with obesity has been described in fragile X syndrome individuals with the CGG expansion mutation [29], indicating that the obesity is probably due to loss of *FMR1* function and interaction with other genes. In addition to these deletions, 4 duplications [30-33], 2 inversions [34,35] and 1 translocation [36] involving the X chromosome have been reported to be associated with obesity.

In Table **2**, we summarized reports from the literature in which structural chromosome abnormalities were reported with syndromic obesity [20-102]. Those involving chromosome 15q11.2 and Prader-Willi syndrome were excluded as this genetic obesity syndrome will be described separately in this journal issue. The most frequently reported chromosome regions were 15q (not shown), 6q [58-71] and Xq [20-36]. Single-minded 1 (*SIM1*) gene (chr.6q16.3, position:100,836,750-100,911,551; hg19) mutations are one of the few known causes of nonsyndromic and PWS- like monogenic obesity in both humans and mice. 

The mouse *Sim1* gene is expressed in the developing kidney and central nervous system and essential for formation of the supraoptic and paraventricular (PVN) nuclei of the hypothalamus implicated in the regulation of body weight. The melanocortin-4 receptor (*MC4R*) gene is expressed in these brain regions and are physiologic targets of alpha-melanocyte-stimulating hormone which inhibits food intake [103]. Michaud *et al*. [104] also demonstrated that the lethal homozygous *Sim1* (Sim1 -/-) null mutation in mice causes lack of the paraventricular nucleus. However, *Sim1* heterozygotes were viable but developed early-onset hyperphagic obesity with clinical features of metabolic syndrome. A remarkable decrease in hypothalamic oxytocin (Oxt) and PVN melanocortin 4 receptor (Mc4r) mRNA was also demonstrated in conditional *Sim1* homozygous and germ line *Sim1* heterozygous mutant mice suggesting that hyperphagic obesity may be attributable to changes in the leptin-melanocortin-oxytocin pathway [105].

Other genes such as *MC4R*, leptin and leptin receptor, Ghrelin (*GHRL*), peroxisome proliferator-activated receptor gamma (*PPARG*), oxytocin receptor (*OXTR*), prohormone convertase-1 and proopiomelanocortin have been implicated in human obesity and will be discussed elsewhere in this journal issue. Heterozygous or bi-allelic disruptions of these genes by structural chromosome abnormalities may potentially lead to obesity. For example, Bittel *et al*. [50] reported a boy with marked obesity and a duplication of chromosome 3p25.3-p26.2 region which contains *GHRL* and *PPARG*. They reported increased expression of these genes which appears to contribute to the obesity seen in this individual. In most of the remaining chromosomal abnormalities in individuals with obesity identified in our literature search listed in Table **2**, no specific obesity- related gene was identified. 

In conclusion, obesity is a highly complex multifactorial clinical phenotype with significant genetic predisposition. So far, several single genes and genomic loci scattered on most of the chromosomes except the Y chromosome have been reported and are involved in the pathogenesis of human obesity. The genetic basis of syndromic obesity in our four selected individuals was identified or confirmed using aCGH microarray analysis. Improved understanding of the specific mechanisms of these genetic predispositions will be crucial for the personalized management of individuals with various forms of obesity, particularly in early childhood. 

## Figures and Tables

**Table 1:** Summary of the Clinical and Molecular Findings in the Four Reported Subjects in our Study

Subject	Chromosome abnormality	FISH study/ Probe	Clinical features
1	Chr. 10q22.3-q23.2 dup (81,281,895-89,091,213bp)	Yes/ RP11-830J13	34 yr old, early onset obesity, idiopathic urticaria, endometriosis, atrophy & scarring of right kidney
2 and 3	Chr. 16p11.2 del (28,730,299-29,009,896 bp)mat	Yes/ RP11-1136I3	Early onset obesity, developmental and speech delays
4	Chr. Xq27.1-q28 del (139,354,859-150,046,723 bp)dn	No	Early onset obesity (truncal), hypotonia, microcephaly, global developmental delays, right esotropia, short stature

**Table 2:**
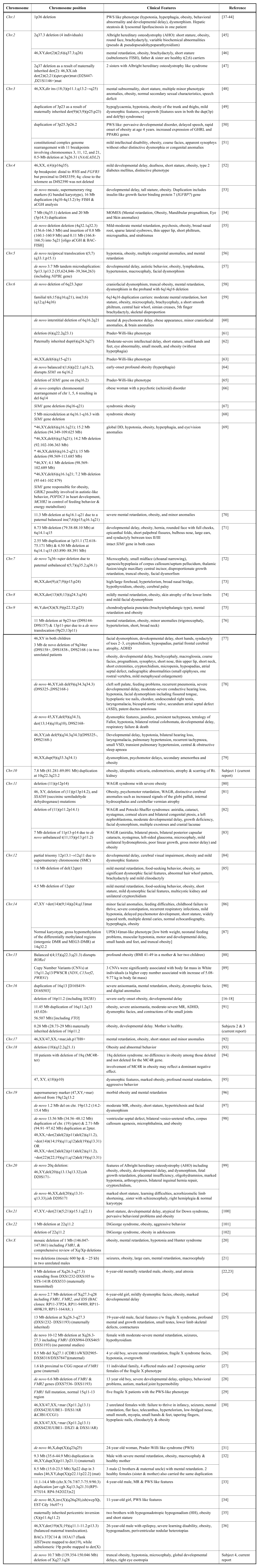
Summary of Reported Chromosomal Abnormalities in the Literature from Individuals with Syndromic Obesity excluding Prader-Willi Syndrome

## WEB RESOURCES

The Center for Applied Genomics (TCAG): http://www.cag.icph.org/

Database of Chromosome Genomic Variants (http://projects.tcag.ca/variation)

miRBase: the microRNA database: http://www.mirbase.org/
